# Detection Methodologies for Pathogen and Toxins: A Review

**DOI:** 10.3390/s17081885

**Published:** 2017-08-16

**Authors:** Md Eshrat E. Alahi, Subhas Chandra Mukhopadhyay

**Affiliations:** Department of Engineering, Macquarie University, Sydney, NSW 2109, Australia; subhas.mukhopadhyay@mq.edu.au

**Keywords:** smart sensors, pathogen, toxin, endotoxin, bacterial infection, chemical sensors, biosensors

## Abstract

Pathogen and toxin-contaminated foods and beverages are a major source of illnesses, even death, and have a significant economic impact worldwide. Human health is always under a potential threat, including from biological warfare, due to these dangerous pathogens. The agricultural and food production chain consists of many steps such as harvesting, handling, processing, packaging, storage, distribution, preparation, and consumption. Each step is susceptible to threats of environmental contamination or failure to safeguard the processes. The production process can be controlled in the food and agricultural sector, where smart sensors can play a major role, ensuring greater food quality and safety by low cost, fast, reliable, and profitable methods of detection. Techniques for the detection of pathogens and toxins may vary in cost, size, and specificity, speed of response, sensitivity, and precision. Smart sensors can detect, analyse and quantify at molecular levels contents of different biological origin and ensure quality of foods against spiking with pesticides, fertilizers, dioxin, modified organisms, anti-nutrients, allergens, drugs and so on. This paper reviews different methodologies to detect pathogens and toxins in foods and beverages.

## 1. Introduction

A significant concern all over the world is food safety. There are a lot of foodborne microorganisms which are responsible for foodborne diseases, which occur due to the contamination of food contaminated by such organisms as fungi, bacteria, and viruses . These are called foodborne pathogens and are responsible for poisoning food and water [1]. There are certain bacterias which are leading causes of foodborne diseases; among them *Escherichia coli*, *Salmonella enterica [2]*, *Campylobacter jejuni*, *Staphylococcus aureus*, *Listeria monocytogenes*, and *Bacillus cereus* are more important. In recent times, a significant number of people have died due to foodborne diseases, and such pathogens are a priority and a critical health issue all over the world [3]. It is challenging to assess foodborne diseases on a global scale, but one report [4] said that roughly one of every six Americans in the U.S.A. (or forty-eight million people) get sick, the number of hospitalized patients is 128,000, and 3000 people die due to foodborne diseases annually. The high rate of foodborne diseases in many emerging countries presents major food safety concerns and difficulties; therefore, it is important to detect the responsible pathogens to reduce foodborne diseases. People who suffer foodborne diseases show some symptoms, such as vomiting, nausea and a disrupted nervous system, and these symptoms may occur in a short period or within 48 h, indicateing the seriousness of the contamination. All of the symptoms may affect one person or a number of people from a community, eventually called an outbreak.

It is hard to estimate the foodborne illness occurring due to different microbial hazards. However, it is seen from Table 1 [5] using estimates from the WHO [6,7], USA [8], Canada [9], Australia [10,11], England and Wales [12], and The Netherlands [13]. The major challenge of foodborne diseases is infection by bacteria [14]. There are two groups of bacteria, named Gram-positive and Gram-negative bacteria, and the difference between them is in the cell structure of their wall. Nearly 95% of pathogenic bacteria are Gram-negative bacteria, and the rest are Gram-positive bacteria [15].

The toxins that are produced by the pathogens are required to be monitored for food safety. Some toxins produced from common organisms, such as *Staphylococcus aureus*, grow on foods and produce Staphylococcal enterotoxin A and B which cause a form of food poisoning. *E. coli* O157: H7 produces Shiga-like toxins 1 and 2 that causes dysentery, haemorrhagic colitis, and haemolytic uremic syndrome. *Listeria monocytogenes* produces an exotoxin called listeriolysin O that has haemolysin functionality. Lipopolysaccharides (LPS) are also considered to be hazardous toxins, and irritants from the outer membrane of Gram-negative bacteria including cyanobacteria are called endotoxins [16]. The conditions under which the toxins are expressed are relatively rare, and further studies are required in this field [17].

There are some traditional methods available for pathogen and endotoxin detection which depend on culturing the microorganisms on agar plates. All the conventional methods are laborious and take 2–3 days to get initial results. It takes up to one week to determine the specific pathogen microorganisms. Therefore, different methods of pathogen detections are in high demand in the food industry to avoid the spread of any bacterial diseases from food poisoning [18,19]. The detection methods can be categorized into the following groups: conventional methods and various sensor-based methods. The objective of this paper is to review these methods of detection and identification and to discuss the advantages, disadvantages and various characteristics of those methods.

## 2. Different Conventional Methods

There are several methods available to detect pathogens and endotoxins from Gram-negative bacteria or to detect the endotoxin itself. Detection of a pathogen will specifically detect the Gram-negative bacteria causing the endotoxin, whereas the detection of endotoxin method detects the structure of lipopolysaccharide (LPS) on the outer membrane of Gram-negative bacteria. The basic chemical structure of LPS consists of four covalently linked segments: a surface carbohydrate polymer, a central oligosaccharide with an inner and outer region and an acylated glycolipid. Different pathogen and endotoxin methods (LPS detection) are discussed in the following sections.

### 2.1. Pathogen Detection Methods

In conventional methods, the detection of pathogens mostly depends on the identification of precise microbiological and biochemical constituents [20]. There are three types of conventional methods: the immunology-based method, the count method of culturing and colony, and the polymerase chain reaction method (PCR) [17,20,21,22]. The culturing technique gives accurate results due to its high selectivity and sensitivity [23,24]. Various microorganisms grow on the food sample and, depending on the growth of the microorganisms, the specific pathogens are identified. The process requires selective plating, pre-enrichment, selective enrichment, and identifications, which take a few days to produce the results. This detection method is monotonous and lengthy.

**Figure 1:**
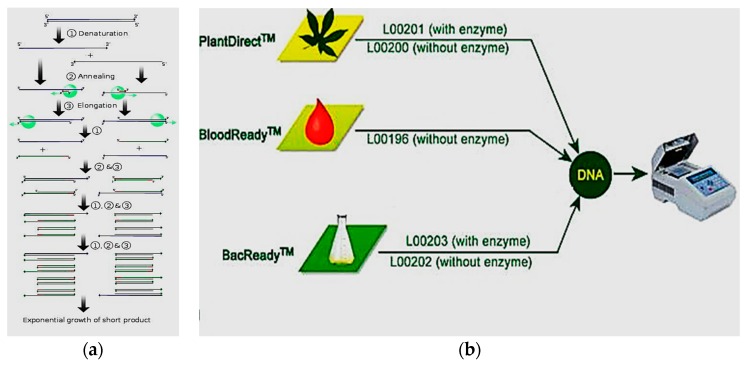
Steps of (**a**) PCR cycle and (**b**) DNA extraction.

The polymerase chain reaction (PCR) is very popular detection method for detection of pathogens [25,26]. Specific bacteria based on their nucleic acid sequence [27,28,29], protozoa [30,31], and viruses [32,33] are targeted when PCR is used for pathogen detection. Different PCR methods are available for pathogen detection, called, reverse transcript PCR (RT-PCR) [34,35], real-time PCR [36], and multiplex PCR [37]. Figure 1 shows a schematic diagram of the PCR cycle to extract the DNA from bacteria for detection of the pathogen. However, the method requires costly instruments, and amplification, isolation, and quantification of DNA technology make it a complex method to perform. It also requires trained personnel to operate the whole procedure.

**Figure 2:**
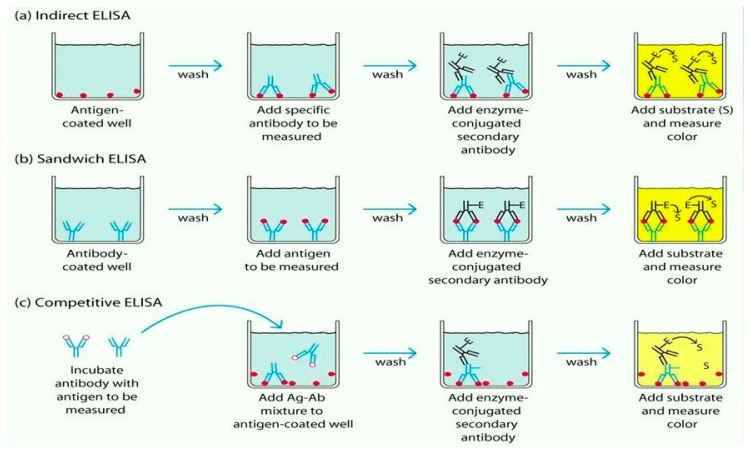
Immunology-based technique using indirect ELISA, sandwich ELISA, and competitive ELISA through schematic diagrams.

The immunological detection technique is used for the detection of pathogens [38,39]. The antigen-antibody bindings are utilized widely in immunological detection for pathogens from Gram-negative bacteria. This method has been successfully used to detect *Salmonella* and *E. coli* [40,41]. Enzyme immunoassay (EIA) [42,43], enzyme-linked fluorescent assay (ELFA) [44,45], enzyme-linked immunosorbent assay (ELISA) [46,47], flow injection immunoassay [48], and other immunological methods [49,50] are mostly used for immunological detection. They require less time to prepare the assay than a culturing technique. However, real-time pathogen detection is not possible with this method [51]. Figure 2 shows a schematic diagram of the immunology-based technique in different ELISA.

### 2.2. Detection Methods of Endotoxin

The rabbit pyrogen test and limulus amoebocyte lysate (LAL) [52,53] are used to detect endotoxins, and are considered conventional methods. The United States Food and Drug Administration (FDA) has approved these methods as the standard method. In 1920, the rabbit pyrogen test was first developed. A test solution is injected into a rabbit’s body and one waits for some time to see any change of the body temperature to detect an endotoxin. However, an animal rights group has opposed using this method, to stop killing rabbits. Additionally, the method is time-consuming and expensive. Now the method is losing favour, and only the LAL test is used, first discovered 67 years ago in 1950 by Dr Frederik Bang [54]. He first observed that horseshoe-crab blood forms clots when it is exposed to endotoxins.

Endotoxin-contaminated food or water is mixed with horseshoe-crab blood to derive the amoebocyte extraction, than one waits to watch for any response from endotoxins. The FDA has approved four tests: chromogenic assay, colorimetric [26,55] (lower protein), gel-clot, and turbidity metric (spectrophotometric). The particular reaction of amoebocyte/endotoxin has characterized these methods. The method of gel clots is based on the existence or nonexistence of gel formation in the sample: when endotoxins are present in the sample gelation occurs due to the coagulation of protein. Turbidity occurs due to the sharp division of an endotoxin, sensitive substrate, and the turbidimetric methods use this turbidity to detect endotoxins. There is another technique which is called the chromogenic technique, depending on the change of colour during the division of a complex into a peptide and a chromogen [56].

The concentration of Lipopolysaccharide (LPS) is expressed as EU/mL or EU/mg, where EU stands for endotoxin unit for biological activity in LPS. Suppose that in one EU, 10^−15^ g of LPS is contributed by Gram-negative bacteria. Therefore, at most 10^5^ bacteria can be generated. The response of LAL test is quick and takes approximately 30 min to get the result. The detection limit is quite low, and the technique is highly sensitive compared to other detection methods. The major disadvantage is that it requires expert personnel to complete all the complex steps to avoid any external interference. Another disadvantage is that the testing kits are expensive for some sampling tests.

## 3. Biosensor-Based Method

The sensor measures physical and chemical quantities and converts them to an electrical signal. Sensors are a kind of transducer where they change one form of energy into another kind of energy. The sensor can measure much physical activity, such as temperature, humidity, acceleration, distance, and many more things. A biosensor is a device which measures living organisms or biological molecules, to detect chemicals present in living organisms.

The recognition is achieved by finding a molecular species to create a binding with the target pathogen for sensing. Different bioreceptors have been introduced in biosensors to increase the efficiency of the measurement. Bioreceptors play an important role in biosensor development, and different types of bioreceptor are discussed in the following section.

### 3.1. Different Types of Bioreceptor

It is important to design the specificity of a biosensor, and bioreceptors play a major role. They are responsible for binding the analyte to the sensor for measurements. Bioreceptors are categorized as: (1) antigen/antibody; (2) enzymes; (3) nucleic acids/DNA; (4) cellular structures/cells; (5) biomimetic; and (6) bacteriophage. Some of them are discussed below.

**Figure 3:**
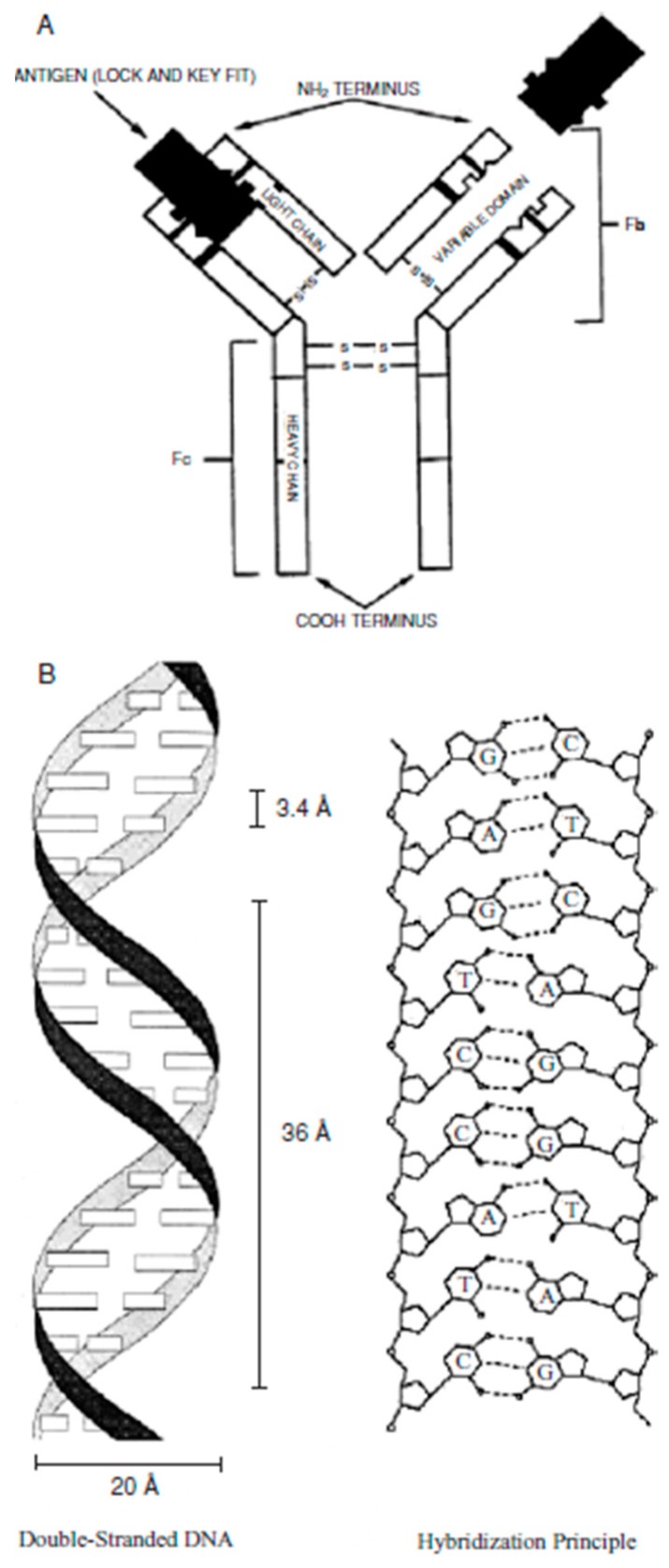
Schematic of two different bioreceptors: (**A**) antigen and (**B**) nuclei acid [68].

Enzyme-based bioreceptors involve enzymes which have specific bindings. All the enzymes are from the protein group except a small group of the catalytic ribonucleic molecule [56,57]. An antibody/antigen is a complex molecule, which is made up of hundreds of individual amino acids arranged in a highly ordered sequence. They have a particular binding ability for a specific structure, which is used as a bioreceptor [58]. A biomimetic-based bioreceptor is an artificial receptor that is designed and fabricated to mimic a biological receptor. Molecular imprinting, plastic membrane fabrication, and genetic engineering are used to design biomimetic-based bioreceptors [59]. An entire cell/microorganism of biorecognition, or a specific cellular component, is capable of creating a specific binding, which is used in cellular based bioreceptors [60], and nucleic acid/DNA based bioreceptor forms of the complementarity of adenine:thymine (A:T) and cytosine:guanosine (C:G) pairings inside the DNA, which ensures the specificity of the biorecognition [61,62]. Figure 3 shows schematic of antigen and nucleic-acid based bioreceptor.

**Figure 4:**
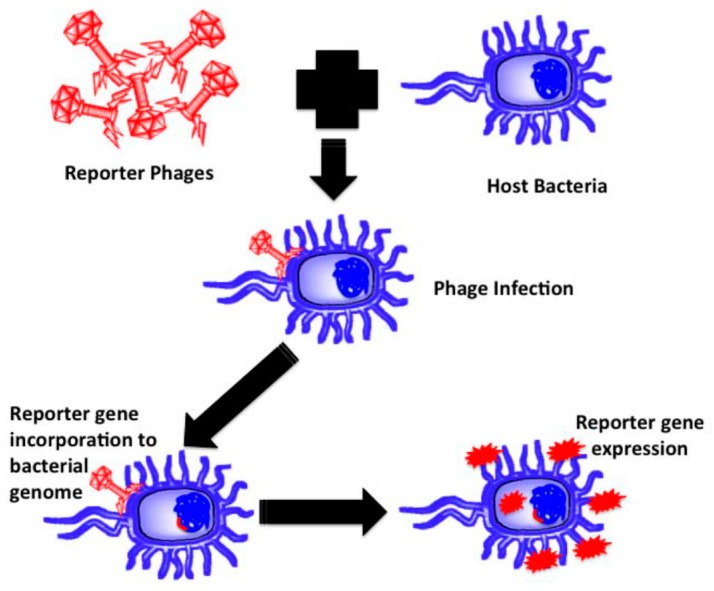
Detection of a pathogen by the phage-based method [69].

There are some obligate parasites, named phages, that do have no organism for metabolic purposes. They depend on their host bacteria for growth and propagation to become mature. Most of them know their specific host bacteria, whereas, a few of them create binding and killing within an entire bacterial genus [63]. Similar to other parasites, phages’ DNA has to be injected into the host bacteria to bind and take over the bacterial organisms (host) to the number of virions. The host DNA is occupied by the genome of the virions, and the genomes remain inactive temporarily until stimulated for propagation. Much research has reported on phage based biosensors for pathogen and endotoxin detection. Various analytical methods combine with bacteriophage-based probes to detect specific recognition sites. Some pathogens are detected in fresh milk [64,65], fresh tomato [66], and water [67], by using phage-based biosensors directly and efficiently. Figure 4 shows the basic steps of pathogen detection using a phage biosensor.

**Figure 5:**
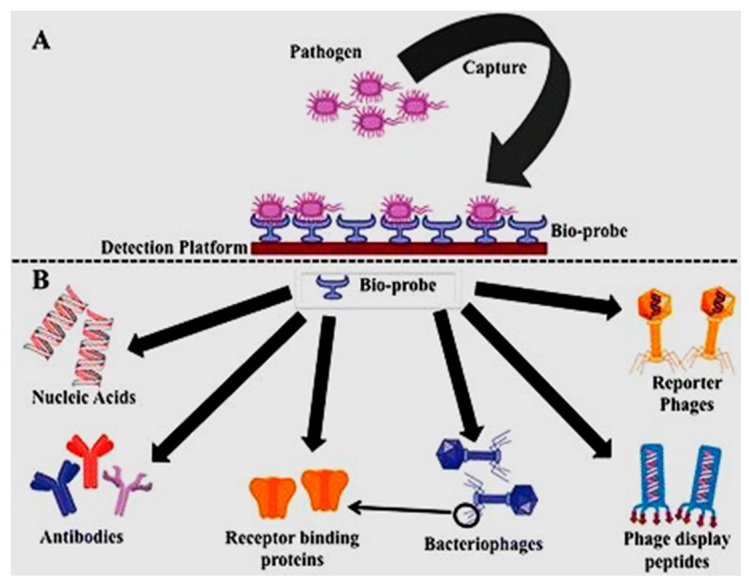
(**A**) Capturing the pathogen on the sensing platform; and (**B**) varieties of bio-probe surface for pathogen detection [70].

Figure 5 shows pathogen binding activity through different bioreceptors, used to design the specificity of the target pathogens on the transduction surface of the biosensors.

### 3.2. Types of Biosensing Methods

**Figure 6:**
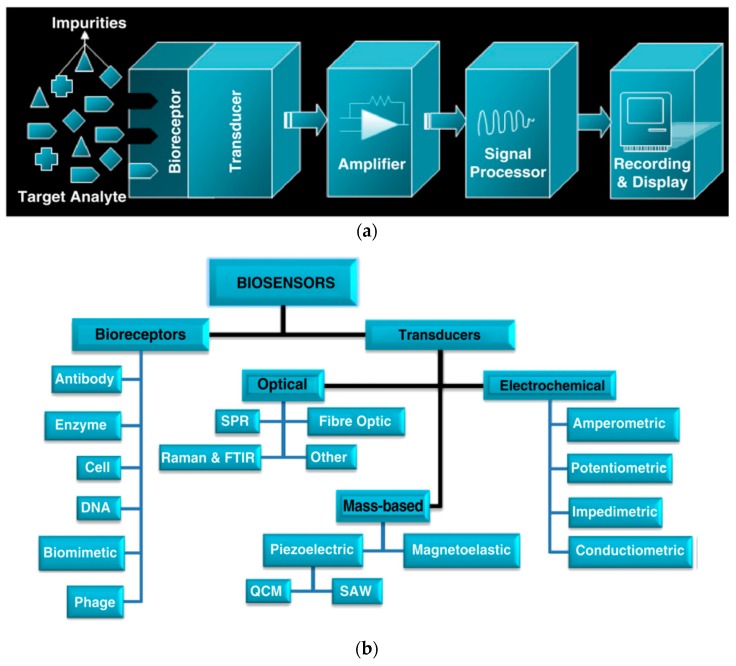
(**a**) Stages of a biosensor; and (**b**) classification of biosensors for different transducers [20].

In the food industry, different biosensing methods are developed to detect pathogens, and have been widely used for some time [14,17,20]. Among all the methods, optical-, electrochemical-, and mass-based transduction are the sensitive and accurate methods. In biosensor application, a bioreceptor surface is required for recognition purposes to specify different pathogens [20]. Figure 6 shows the different stages of biosensing methods and their classification in terms of sensing methods.

#### 3.2.1. Biosensors Based on the Optical Method

This method is very successful and reliable to detect pathogens and endotoxins [71]. The basic optics characteristics such as reflection, refraction, absorption, dispersion, etc., are used to develop optical biosensors. Optical fibre, Raman infrared spectroscopy, surface plasmon resonance, and others are used as transducers to develop optical biosensors [71].

Laser light propagates through the tapered optical fibre on the detection surface and then that emitted light is detected. The propagated light goes through the fibre or waveguide and detects foodborne pathogens and different endotoxins. These methods were used to develop optical-based biosensors, to detect pathogens such as *E. coli* [72,73], *Salmonella* [74,75], *Listeria* [76,77], and others [78,79]. Su et al. [80] and Marazuela et al. [81] have reported the detection of endotoxins where an optical-based biosensor was used as a detection method.

**Figure 7:**
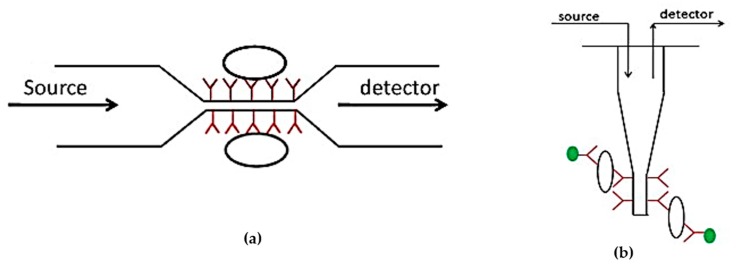
(**a**) Schematic diagram of a tapered optical biosensor; (**b**) Schematic diagram of a tapered tip optical biosensor [89].

Reflectance spectroscopy is used to detect pathogens and endotoxins for surface plasmon resonance (SPR). SPR measures the changes of the reflected angle of the light when the cells create binding on the specific receptor [82]. Wang et al. [83], Torun et al. [84], and Zhang et al. [85] have reported many studies on pathogen and endotoxin detection based on SPR methods. There are other pathogens, and endotoxin detection methods are available, such as optical micro-ring resonators [86], Raman and FTIR spectroscopy [87], and fluorescence detection [88]. Figure 7 shows optical methods to develop a biosensor for pathogen detection.

#### 3.2.2. Biosensors Based on the Electrochemical Method

**Figure 8:**
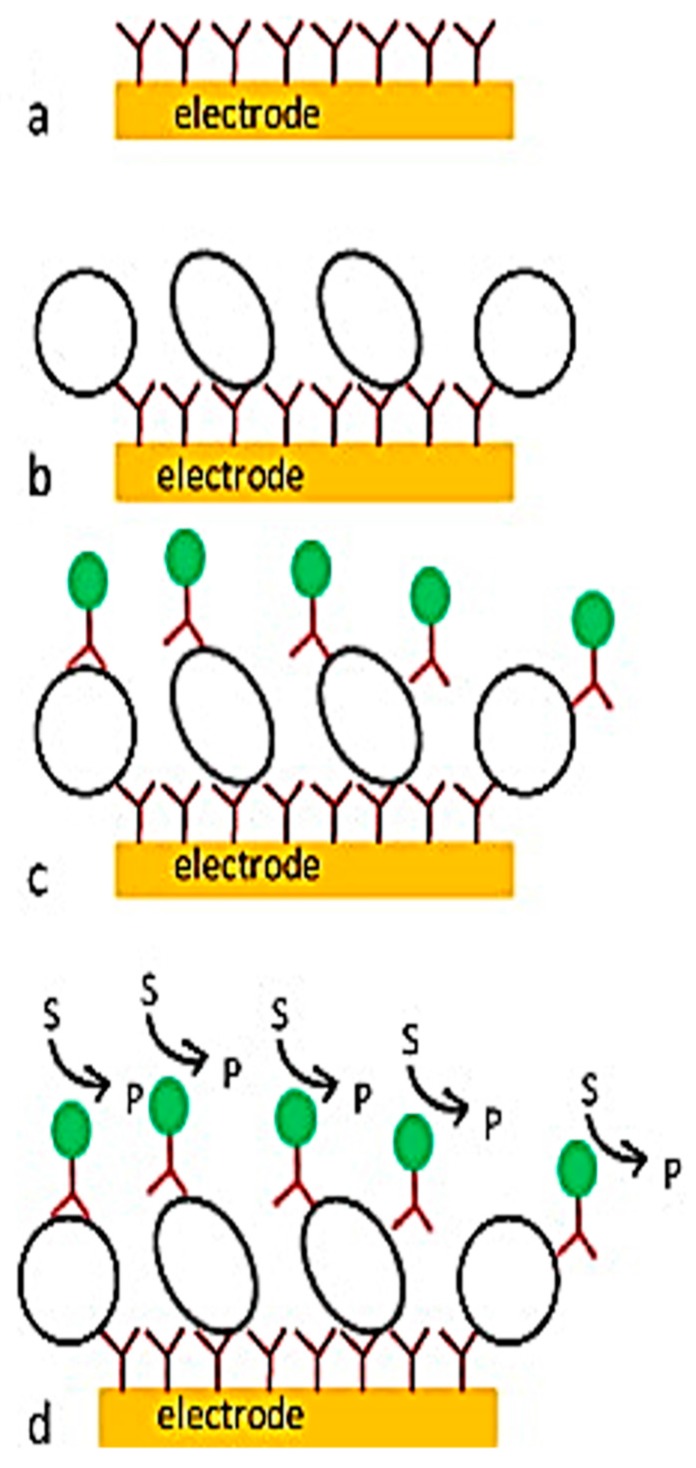
Schematic diagram of an electrochemical biosensor. (**a**) Recognition molecules are immobilized on the electrode surface; (**b**) antigen bonds with the recognition element; and (**c**,**d**) electrical signal is generated due to the redox reaction in between the binding antigen and the recognition element [89].

When the sensing electrode interacts with the sample, the changed potential and current are observed; this biosensor is called electrochemical biosensors [90]. Different types of electrochemical biosensors are available, such as the potential (potentiometric), current (amperometric), conductance (conductometric), and impedance (impedimetric) types [91,92,93]. The advantages of this biosensor are that it is low cost, miniature and robust to liquid samples. Compared to optical biosensors, the selectivity and sensitivity are a little restricted, but their use coupled with other bio-sensing methods can increase the performance of detection [20]. Figure 8 shows the schematic diagram of an electrochemical sensor and the steps enabling the pathogens to be detected in real-time.

##### Biosensors Based on the Amperometric Principle

One common type of an electrochemical biosensor for detection of pathogens is based on the amperometric principle. In this method, a current flow is measured which relates to the concentration of a measured analyte, such as a pathogen. The sensor based on amperometric principle applies a constant value of potential difference between the electrodes, and the resulting flow of current is measured [94]. The applied potential drives the movement of electrons and the rate of flow is measured. The amperometric-based biosensor has been used to detect *E. coli* [95,96], *Salmonella* [97], and others [98]. Usually, it offers improved sensitivity compared to the method based on the potentiometer. The developed method has been used to detect specifically *E. coli*, *Mycrobacterium smegmatis*, and *Bacillus cereous* has been reported in [99].

##### Biosensors Based on Impedimetric Measurement

In this type of biosensor, the impedance of the sensor is measured, which is affected by biological reaction. In recent years, the impedance-based biosensor has attracted widespread attention [100]. Electrochemical impedance spectroscopy (EIS) has a significant role in biosensor-based impedance measurement. EIS is usually used to determine the electrical properties of any material along with their interfaces using surface-modified electrodes [101]. A small alternating voltage is applied across the electrode terminals over a wide frequency range. The current between the electrodes passes through the material under observation. The impedance is measured and is then analysed using the EIS technique. The method has been used to detect pathogens [100,102,103,104,105]. In Wang et al. [106] have reported the new trend of biosensors based on impedance measurement. Detection of endotoxin has been reported in [80]. Pathogens and phthalates in water have been detected using the method and are reported in [107,108,109,110,111,112,113].

##### Biosensors Based on the Potentiometric Principle

In the potentiometric biosensor, the outcome of the bio-recognition activity is converted into a potential signal. The method uses an ion-sensitive field-effect transistor (ISFET) or ion-selective electrodes (ISE), where the ions accumulate at the interface of the ion-selective membrane and produce the primary output signal. In this method, the amount of current flowing through the electrodes is approximately zero. Since enzyme reaction ions are produced, which results in a potential across the electrodes. Research on biosensors for pathogen detection using this principle are reported in [114,115,116].

#### 3.2.3. Biosensors Based on Mass-Sensitivity

Biosensors including the quartz crystal microbalance (QCM) and surface acoustic wave (SAW) belong to this category.

##### QCM-Based Biosensors

A QCM [117] based biosensor operates on the piezoelectric principle. An electric signal is applied between two gold plates on a quartz crystal to produce a vibration at a specific resonance frequency [118], which is measured. When the QCM is used as a biosensor, the resonance frequency of the crystal changes in the presence of the measurand, as it changes the mass of the crystal. The shift in frequency of the crystal is measured. The QCM-based biosensor has been used to detect pathogens [119] and endotoxins [120].

##### SAW-Based Biosensor

The surface acoustic wave (SAW) based interdigital transducer [IDT] [121] produces acoustic waves on the surface from the piezoelectric substrate and changes due to the measurand are detected. Acoustic waves are generated and detected on the piezoelectric surface using IDTs. SAW-based biosensors have been used for various applications including for detection of pathogens and endotoxins. Rocha-Gaso et al. [122] reported pathogen detection and Lange et al. [82] and Hammer et al. [123] have reported endotoxin detection by this method.

Table 2 shows the different detection methods and summarizes the response time and limit of detection for *E. coli* as one of the important pathogen. From Table 2, it is also seen that the biosensing methods are fast responding and reliable for in situ real-time monitoring.

## 4. Nanomaterial-Based Sensors

In recent years, great advances have been made in nanomaterial-based biosensors, where the sensing electrode is modified by a nanomaterial to achieve a quick electron transfer due to the stimulation of different biomarkers. Due to this advantage, much research has been reported where the nanomaterials are coupled with biomolecules to develop nanomaterial-based biosensors to detect dangerous pathogens. *Salmonella*, *E. coli* [137], and *L. monocytogenes* are the most studied pathogens, developing their detection methods in food. However, the respiratory syncytial [138,139] virus and parasites (*Giardia* and *Cryptosporidium*) [140] are other pathogens which are responsible for contaminating food. Yang et al. [141] reported a sensor to detect *Salmonella spp.*, where gold (Au) nanoparticles were used on a glassy carbon electrode. The sensing surface was selective for *Salmonella spp.* With the increased sensitivity and selectivity. Nanowire-based sensors have attracted great interest in recent years. Wang et al. [141] developed TiO_2_ nanowire microelectrodes for rapid and sensitive detection of *Listeria monocytogenes*. This method has an excellent stability, a low detection limit, and can avoid interference from other foodborne pathogens. Reduced graphene sheets (RGSs) contain a unique nanostructure, which is similar to a solid honeycomb crystal structure with excellent electrical conductivity, chemical stability, and mechanical strength. Wan et al. [142] developed an RGS-based sensor which can detect sulphate-reducing pathogens without the obvious response to *Vibrio angillarum* with excellent sensitivity, selectivity, stability, and good analytical performance. Additionally, nanopore-material-based sensors have shown a unique advantage for pathogen detection. Wang et al. [143] immobilized ssDNA which is functionalized for *E. coli* O157: H7 on an aluminium anodized oxide nanopore membrane.

**Figure 9:**
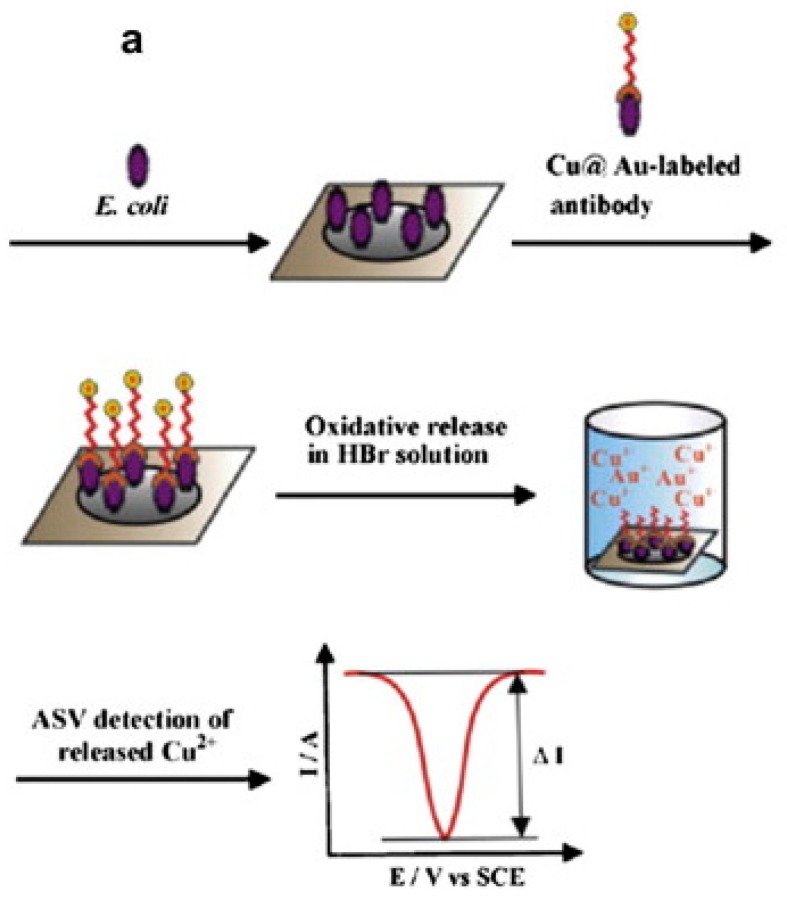
Steps of nanomaterial-based biosensing of pathogen.

Therefore, nano-materials have been used to develop a cost-effective biosensor for in situ measurement. Some more nanomaterial-based biosensor have been reported where nanoribbons [144], nanowires [145], carbon nanotubes [146,147,148], etc., are used to manufacture the biosensor for pathogen detection. The nanomaterial’s surface bonds with the recognition element to develop a biosensing platform, and their combined mechanism generates signal transduction for the monitoring of pathogens in food or water. Figure 9 shows the steps of pathogen detection.

Table 3 shows different biosensors with their advantages, limitations, and cost.

## 5. Conclusions

Detection of pathogens and toxins at an early stage is crucial to avoid food poisoning and other problems. There are different methods available for their detection. Conventional methods are mainly laboratory-based and usually take a long time to detect pathogens and toxins. Many new approaches, such as electrochemical-, optical-, and nanomaterial-based biosensors have been developed. Each developed method has its advantages and disadvantages. The adopted method should be reliable, accurate, and selective to a particular pathogen/toxin, as well as fast enough to obtain reliable results. The paper has reviewed different methods along with the sensors for detection available to scientific communities.

## Figures and Tables

**Table 1:** The estimated foodborne illness due to different pathogens from a few countries [5].

Hazard	Percentage Foodborne (%)					
	WHO (2015) ^a^	USA (2011)	Canada (2015)	Australia (2005, 2014)	England and Wales (2002)	Netherlands (2008)
**Bacteria**						
*Bacillus cereus*	100	100	99	100	100	90
*Campylobacter* spp.	51–76	80	62	77 ^b^	80	42
*Clostridium perfringens*	100	100	93	98 ^b^	94	91
Shiga-toxin-producing *Escherichia coli*(STEC) O157:H7	40–60 ^c^	68	61	56 ^b,c^	63	40
STEC non-O157	40–60 ^c^	82	60	56 ^b,c^	63	42
*Listeria monocytogenes*	100	99	77	98 ^b^	99	69
*Salmonella* non-typhoidal	46–76	94	63	72 ^b^	92	55
*Shigella* spp.	7–36	31	26	12 ^b^	8	NE
*Staphylococcus aureus*	100	100	78	100	96	87
*Yersinia enterocolitica*	NE	90	83	75	90	NE
**Parasitic**						
*Cryptosporidium parvum*	8–16	8	11	10	6	12
*Giardia lamblia*	1–14	7	7	5	10	13
**Viruses**						
Hepatitis A virus	2–42	7	30	12 ^b^	11	11
Norovirus	1–26	26	18	18 ^b^	NE	17

^a^ = The WHO study estimated for 14 international regions; ^b^ = Marked estimation is from 2014. Rest are from their earlier publication; ^c^ = Estimate was derived for total STEC; NE = not estimated.

**Table 2:** Existing *E. coli* method of detection, sampling, and their response summary.

Name of the Method	Sample	Limit of Detection (CFU mL^−1^)	Response Time (Approx.)	Reference
**Conventional Methods**				
Culturing	Apple cider	Low CFUs	1 day to 7 days	[124]
Fluorescence-based bacteriophage assay	Broth	10.00–100.00	10 h	[125]
Capillary-based immunoassay	Ground beef and apple cider	0.5–1.00	7 h	[126]
Fluorescence-based immunoassay	Apple cider	10.00–100.00	6 h	[127]
ELISA	Ground beef, pork, turkey, Fermented sausage, salad, oriental salad and sausage	1.20 × 10^3^	1 day	[128]
ELISA based PCR	Milk	100.00	5 h	[129]
Real-Time based PCR	Ground beef	5 cell	5 h 20 min	[130]
**Biosensing Methods**				
Fibre Optic-based Immunosensor	Broth	2.90 × 10^3^	10 h	[131]
SPR	Milk	10^2^	60 min	[132]
QCM-based Immunosensor	Cow’s preputial washing and vaginal mucus	10^3^	170 min	[133]
Amperometric	Solid Food	100.00–600.00	30 min	[134]
Conductimetric	Water	79.00	10 min	[135]
Impedimetric	Romaine lettuce wash water	10^4^ in culture and 10^7^ in water	10 min	[136]

**Table 3:** Summary of various biosensors with their advantages and limitations.

Method of Detection	Advantages	Limitations	Cost	References
Optical methods	Sensitivity is high, nearly can detect in real time and detection system is label-free	Cost is very high	High	[149,150]
Electrochemical methods	Requires large quantity of sample numbers, might be automatic and detection system is label-free	Specificity is low and not suitable for low sensitivity and needs a lot of washing steps	Low	[149,150]
Mass-based methods	Cheaper than other methods, easy operation, can be able to detect in real-time; moreover, detection is label-free	Specificity and sensitivity are low, requires long incubation time and problematic to regenerate the crystal surface	Low	[149,150]
Nanomaterial-based Sensors	User-friendly measurement and measurement can be done in real time	Concerns are there regarding the toxicity of the nanomaterial and may not be possible to regenerate the sensor	Medium	[151]
